# Primary ChAdOx1 vaccination does not reactivate pre-existing, cross-reactive immunity

**DOI:** 10.3389/fimmu.2023.1056525

**Published:** 2023-01-31

**Authors:** Larissa Henze, Julian Braun, Lil Meyer-Arndt, Karsten Jürchott, Maike Schlotz, Janine Michel, Marica Grossegesse, Maike Mangold, Manuela Dingeldey, Beate Kruse, Pavlo Holenya, Norbert Mages, Ulf Reimer, Maren Eckey, Karsten Schnatbaum, Holger Wenschuh, Bernd Timmermann, Florian Klein, Andreas Nitsche, Claudia Giesecke-Thiel, Lucie Loyal, Andreas Thiel

**Affiliations:** ^1^ Si-M/”Der Simulierte Mensch” a science framework of Technische Universität Berlin and Charité - Universitätsmedizin Berlin, corporate member of Freie Universität Berlin, Humboldt-Universität zu Berlin, Berlin, Germany; ^2^ Regenerative Immunology and Aging, BIH Immunomics, Berlin Institute of Health, Berlin, Germany; ^3^ NeuroCure Clinical Research Center, Charité – Universitätsmedizin Berlin, corporate member of Freie Universität Berlin, Humboldt-Universität zu Berlin, and Berlin Institute of Health, Berlin, Germany; ^4^ Department of Neurology with Experimental Neurology, Charité – Universitätsmedizin Berlin, corporate member of Freie Universität Berlin, Humboldt-Universität zu Berlin, and Berlin Institute of Health, Berlin, Germany; ^5^ Laboratory of Experimental Immunology, Institute of Virology, Faculty of Medicine and University Hospital Cologne, University of Cologne, Cologne, Germany; ^6^ Highly Pathogenic Viruses, Centre for Biological Threats and Special Pathogens, WHO Reference Laboratory for SARS-CoV-2 and WHO Collaborating Centre for Emerging Infections and Biological Threats, Robert Koch Institute, Berlin, Germany; ^7^ JPT Peptide Technologies GmbH, Berlin, Germany; ^8^ Max Planck Institute for Molecular Genetics, Berlin, Germany; ^9^ German Center for Infection Research (DZIF), Partner site Bonn-Cologne, Cologne, Germany; ^10^ Center for Molecular Medicine Cologne (CMMC), University of Cologne, Cologne, Germany

**Keywords:** SARS-CoV-2, antigen-specific T-cells, cross-reactivity, heterologous vaccination, humoral response

## Abstract

Currently available COVID-19 vaccines include inactivated virus, live attenuated virus, mRNA-based, viral vectored and adjuvanted protein-subunit-based vaccines. All of them contain the spike glycoprotein as the main immunogen and result in reduced disease severity upon SARS-CoV-2 infection. While we and others have shown that mRNA-based vaccination reactivates pre-existing, cross-reactive immunity, the effect of vector vaccines in this regard is unknown. Here, we studied cellular and humoral responses in heterologous adenovirus-vector-based ChAdOx1 nCOV-19 (AZ; Vaxzeria, AstraZeneca) and mRNA-based BNT162b2 (BNT; Comirnaty, BioNTech/Pfizer) vaccination and compared it to a homologous BNT vaccination regimen. AZ primary vaccination did not lead to measurable reactivation of cross-reactive cellular and humoral immunity compared to BNT primary vaccination. Moreover, humoral immunity induced by primary vaccination with AZ displayed differences in linear spike peptide epitope coverage and a lack of anti-S2 IgG antibodies. Contrary to primary AZ vaccination, secondary vaccination with BNT reactivated pre-existing, cross-reactive immunity, comparable to homologous primary and secondary mRNA vaccination. While induced anti-S1 IgG antibody titers were higher after heterologous vaccination, induced CD4^+^ T cell responses were highest in homologous vaccinated. However, the overall TCR repertoire breadth was comparable between heterologous AZ-BNT-vaccinated and homologous BNT-BNT-vaccinated individuals, matching TCR repertoire breadths after SARS-CoV-2 infection, too. The reasons why AZ and BNT primary vaccination elicits different immune response patterns to essentially the same antigen, and the associated benefits and risks, need further investigation to inform vaccine and vaccination schedule development.

## Introduction

The COVID-19 pandemic caused by severe acute respiratory syndrome coronavirus 2 (SARS-CoV-2) still challenges health care systems and the economy globally. SARS-CoV-2 vaccines were developed and approved at unprecedented speed. However, contrary to initial hopes, they failed to induce sterile immunity (1, 2). These first-generation vaccines included lipid nanoparticle-formulated, nucleoside-modified mRNA vaccines BNT162b2 (Comirnaty, BioNTech/Pfizer, in the following abbreviated as BNT) and m-1273 (Moderna) as well as the adenovirus-vector-based ChAdOx1 nCOV-19 vaccine (Vaxzeria, AstraZeneca, in the following abbreviated as AZ), all encoding for the spike glycoprotein (spike) (3, 4). The BNT vaccine was initially approved for a 21-days interval, 2 dose regimen, whereas for the AZ vaccine, three months between the first and the second dose were approved (5). At first, Germany’s authorities recommended AZ only for the younger adults (<60 years) due to lacking data on efficacy in the elderly (6). Following reports of rare cases of vaccine-induced immune thrombotic thrombocytopenia in young individuals in relation to vaccination with AZ, recommendations for the younger were changed to BNT at the end of March 2021, and resulted in a small cohort of young, heterologous vector-mRNA-vaccinated individuals (7–9). This cohort revealed not only good tolerance of heterologous vaccination but also reported both higher antibody titers and higher neutralization capacity against novel variants of concern (VOCs) (10–13). Vaccination-induced cellular immune responses were comparable between heterologous AZ-BNT vaccination and homologous vector-based vaccination, and higher than following homologous AZ-AZ vaccination (13–16). Accordingly, vector vaccines later were recommended to be combined with mRNA also for third and fourth doses (17, 18). Although the efficacy of mRNA-based vaccines remained unmet, their need for deep cooling and challenging production limits their global usage (5, 19). Additional obstacles of both mRNA- and vector-based vaccines are the administration by injection and especially the reduced effectiveness of neutralizing and blocking antibodies against arising VOCs (20). Some of these hurdles may get solved with the development of second-generation nasal vaccines targeting mucosal immunity and shifting the focus from RBD-specific antibodies to strengthening a broader, pan-coronavirus immunity including optimized T cell responses (21–24). Coronaviruses are widespread in the animal kingdom and further spillover to humans can be expected in the future (25). Prior to SARS-CoV-2, four other common cold coronavirus (hCoV) strains (HKU1, OC43, NL63, 229E) circulated with a seasonal pattern among humans. These are responsible for normal colds and, accordingly, ubiquitous cellular immunity to endemic coronaviruses (26–28). We and others could demonstrate that hCoV induced pre-existing pan-coronavirus-reactive immunity provides rapidly responding CD4^+^ and CD8^+^ T cells in blood and mucosa upon SARS-CoV-2 infection or COVID-19 vaccination (29–34). Within spike, due to homology, cross-reactivity focuses on the S2 subunit. Here, a conserved epitope (iCope) within the fusion domain (aa 816–830) accounts for the majority of responsive CD4^+^ T cells and cross-reactive neutralizing antibodies (28, 29, 35, 36). While infection and homologous BNT vaccination has been shown to boost this cross-reactive immunity (29, 37), the capacity of heterologous vaccination to engage cross-reactive pre-existing immunity is unknown. Therefore, here we comprehensively assessed the quantity and quality of immune responses induced in AZ-BNT vaccination and compare it to that induced by homologous BNT vaccination regimen.

## Results

### Kinetics of cellular and humoral responses in heterologous AZ-BNT vaccination

**Figure 1:**
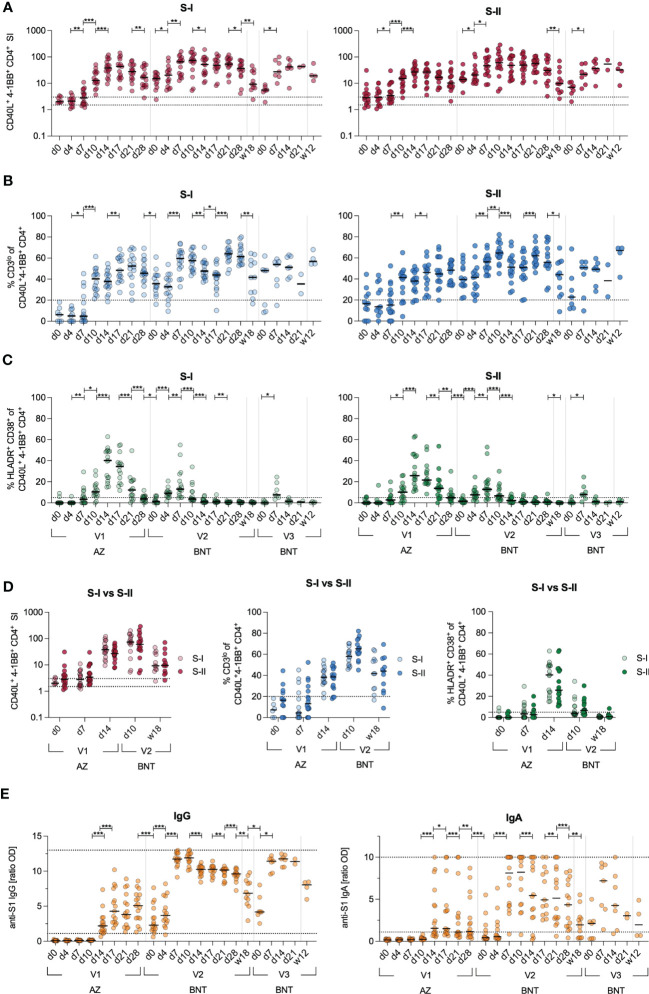
Kinetics of adaptive immune responses upon heterologous vaccination. Immune response kinetics following heterologous SARS-CoV-2 vaccination of unexposed young healthy donors (n=17) vaccinated with AZ at d0, then BNT 85(+/-3) days after first dose and 142(+/-7) days after second dose. **(A)** Ex vivo stimulation of PBMCs with S-I and S-II peptide pools. The percentage of CD40L+4-1BB+ within CD4+ T cells among stimulated PBMCs was divided by the percentage of these cells in the unstimulated control, resulting in the stimulation index (SI). Dotted lines indicate a SI of 1.5 and 3, separating non-responders from responders with uncertainty and definite responders. **(B)** Frequencies of CD3lo cells among S-I- or S-II-reactive CD40L+4-1BB+ CD4+ T cells of T cell responses with a SI ≥ 1.5.  **(C)** Frequencies of HLA-DR+CD38+ among CD40L+4-1BB+ CD4+ T cells. **(D)** Direct comparison of the SI, frequencies of CD3lo and HLA-DR+CD38+ cells of CD40L+4-1BB+ CD4+ T cells at indicated time points upon stimulation with S-I or S-II peptide pools. **(E)** Serum anti-SARS-CoV-2 S1 IgA and IgG titer ratios. Only significant differences are shown with *p < 0.05, **p<0.01, ***p<0.001 (A-C, E-F: Wilcoxon matched-pairs signed-rank test between consecutive days, D: Mann-Whitney test). SI below 1 were excluded from further analysis, as they are below the lower limit of detection. Black line indicates the median.

At first, we examined cellular and humoral response kinetics of 17 donors during heterologous vaccination with a primary dose of AZ and, three months later, a secondary dose of BNT in a 3–4-day sampling interval for the first two weeks and thereafter weekly until day 28 (**Figure S1**). We stimulated PBMCs with a S1 spike peptide pool (S-I) covering the N-terminal amino acid residues 1–643 and a S2 peptide pool (S-II) covering the C-terminal amino acid residues 633–1273. In line with our previous findings (29), antigen-specific CD40L^+^4-1BB^+^ cross-reactive CD4^+^ T cells with high TCR avidity characterized by downregulated CD3 cell surface expression (CD3^lo^) could be observed in response to S-II but not S-I stimulation prior to vaccination (d0) (**Figures 1A, B**). After the second vaccination, both S-I- and S-II-specific CD4^+^ T cells displayed a secondary response kinetics peaking already at d10 with comparable frequencies and TCR avidity which remained stable until week 12 after the third dose of vaccine with BNT (**Figures 1A, B, D**). Utilizing HLA-DR and CD38, we also monitored the proportion of recent *in vivo* activation among S-I- and S-II-specific CD4^+^ T cells in the periphery (**Figure 1C**). The frequencies of HLA-DR^+^CD38^+^ S-I- or S-II-specific CD4^+^ T cells were highest upon the first dose of vaccine and displayed lower frequencies after the second, and particularly following the third dose of vaccine even though the overall frequency of CD40L^+^4-1BB^+^ CD4^+^ T cells were comparable at each peak between S-I and S-II stimulations. (**Figure 1D**). Primary AZ vaccination induced anti-S1 IgG titers in all donors at day 17, but only 66 % (10 of 15) displayed detectable IgA titers above threshold (**Figure 1E**). The second and third vaccine dose increased IgG titers, which however decreased significantly over the observation period of three months. IgA responses were highly heterologous across donors and displayed faster reduction than IgG, already within weeks after the second dose.

### Heterologous vaccination results in slower induction of CD4^+^ T cell responses, but higher IgG responses compared to homologous vaccination

**Figure 2:**
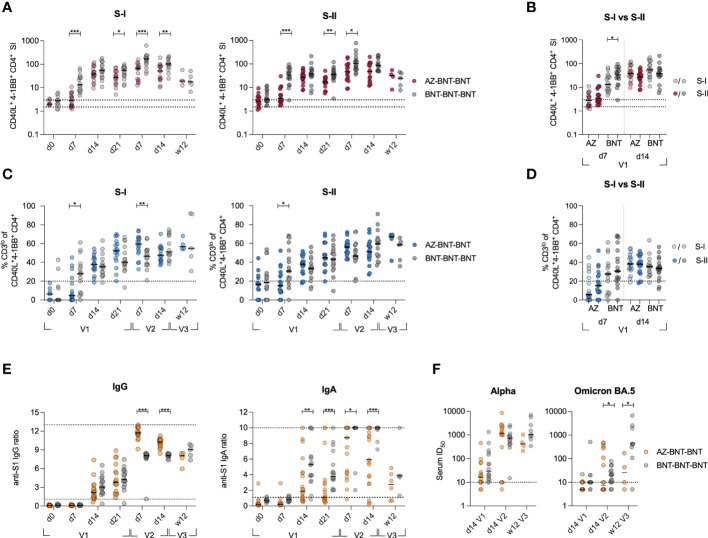
Heterologous vaccination results in slower induction of CD4+ T cell responses, but higher IgG responses compared to homologous vaccination. **(A)** *Ex vivo* stimulation of PBMCs from donors receiving heterologous AZ-BNT and homologous BNT-BNT vaccination with S-I and S-II peptide pools at indicated time points. SI of antigen-specific CD40L+4-1BB+ CD4+ T cells is shown. **(B)** *Ex vivo* stimulation of PBMCs from AZ or BNT primary vaccinated donors with S-I and S-II peptide pools early and at peak of immune response. SI of antigen-specific CD40L+4-1BB+ CD4+ T cells is shown. **(C)** Frequencies of CD3lo cells among S-I- or S-II-reactive CD40L+4-1BB+ CD4+ T cells of T cell responses with a SI ≥ 1.5. **(D)** *Ex vivo* stimulation of PBMCs from AZ or BNT primary vaccinated donors with S-I and S-II peptide pools early and at peak of immune response. Frequencies of CD3lo antigen-specific CD40L+4-1BB+ CD4+ T cells are shown. **(E)** Serum anti-SARS-CoV-2 S-1 IgG and IgA antibody levels (OD) were determined at indicated time points. Upper and lower levels of detection were set at 1 and 13 (IgG)/ 10 (IgA), respectively, indicated by dotted lines. **(F)** Anti-SARS-CoV-2 B.1.1.7 (Alpha) and B1.1.529 (Omicron) subtype BA.5 variant spike neutralizing capacity at d14 post primary, d14 post secondary and 12 weeks post booster vaccination. Positivity thresholds: >10 ID50 for spike neutralization. Serum ID50 values less than the lowest serum dilution tested (1:10) were assigned a value of 5 for plotting the graph and for statistical analysis. Only significant differences are shown with *P < 0.05, **P < 0.01, ***P < 0.001, Mann-Whitney test. SI below 1 were excluded from further analysis, as they are below the lower limit of detection. Black line indicates the median.

Next, we investigated quantitative and qualitative differences in the adaptive responses upon heterologous vaccination and compared them to these of 16 age- and gender-matched donors from a previously published cohort of homologous BNT-vaccinated individuals (29). Note, while the heterologous AZ-BNT vaccination involved a three-month interval between first and second vaccination, the second vaccination in the BNT-BNT cohort was administered three weeks after the first dose. We weekly assessed the response to the first dose, the peak of response after the second dose (d7, d14) and the long-term response at 12 weeks after the third dose of BNT vaccine, administered to both cohorts 6-10 months after the second dose (**Figure S1**). Primary BNT vaccination resulted in a more rapid T cell response, inducing higher frequencies of S-I- and S-II-reactive T cells early at day 7 post primary and after secondary vaccination. This difference, however, vanished three months after the third dose of vaccine (**Figure 2A**). In contrast to BNT-vaccinated, AZ-primed individuals did not display higher frequencies of S-II-specific than S-I-specific T cells early (d7) after primary vaccination (**Figure 2B**). The population’s TCR avidity increased faster following first BNT vaccination in both cohorts, but remained comparable thereafter (**Figures 2C, D**). Upon secondary BNT vaccination, AZ-primed individuals displayed higher IgG levels, but lower IgA levels compared to homologous vaccinated (**Figure 2E**). These differences leveled out three months after the third dose of vaccine. Neutralization against the Alpha variant was achieved as early as 14 days following primary vaccination with AZ or BNT and remained comparable following secondary and tertiary vaccination with BNT (**Figure 2F**). However, neutralization of the Omicron variant was largely absent following primary vaccination and significantly higher following homologous BNT vaccination (**Figure 2F**).

### Lack of evidence for reactivation of cross-reactive CD4^+^ T cells after primary AZ vaccination

**Figure 3:**
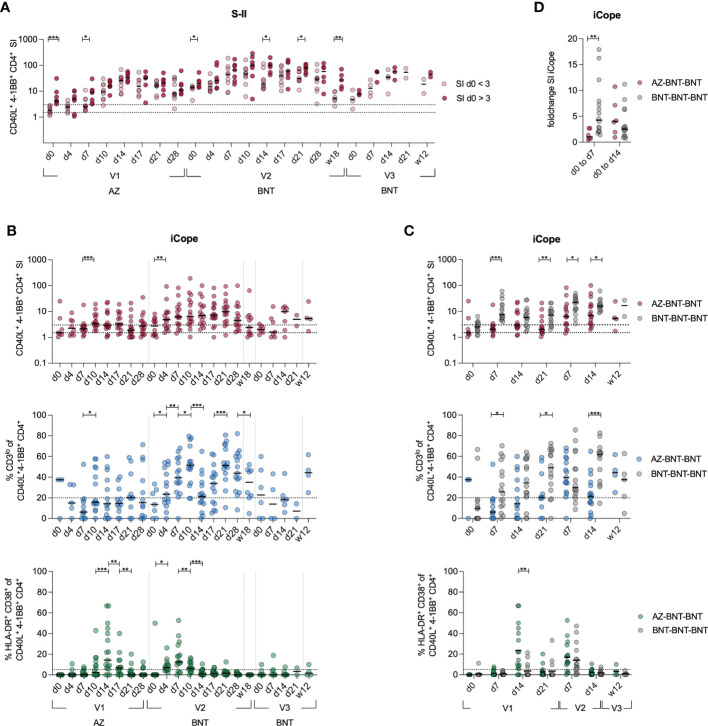
Cross-reactive cellular responses are not effectively induced by AZ primary vaccination. **(A)** *Ex vivo* stimulation of PBMCs with S-II peptide pool. Donors were separated into cross-reactive responders according to an SI of >3 at d0 and non-cross-reactive responders (baseline SI <3). **(B)** Stimulation index of antigen specific CD40L^+^4-1BB^+^ CD4^+^ T cells, frequencies of CD3^lo^ cells among iCope-reactive CD40L^+^4-1BB^+^ CD4^+^ T cells of T cell responses with a SI ≥ 1.5, and HLA-DR^+^CD38^+^ among iCope-reactive CD4^+^ T cells are shown. **(C)** Comparison of SI, CD3^lo^ and HLA-DR^+^CD38^+^ between heterologous (AZ-BNT-BNT) and homologous (BNT-BNT-BNT) vaccinated donors.**(D)** Foldchange of the SI of iCope-specific T cells from d0 to d7 and d0 to d14. Only significant differences are shown with *P < 0.05, **P < 0.01, ***P < 0.001. A, C-D: Mann-Whitney test B: Wilcoxon matched-pairs signed-rank test. SI below 1 were excluded from further analysis, as they are below the lower limit of detection. Black line indicates the median.

To investigate the recruitment of cross-reactive T cells in the immune response following heterologous vaccination, we longitudinally compared the S-II-specific CD4^+^ T cell response from donors with cross-reactive (SI>3 at baseline) CD4^+^ T cells to donors without cross-reactive CD4^+^ T cells (SI<3 at baseline) (**Figure 3A**). Overall, S-II-specific CD4^+^ T cell frequencies remained higher in the cross-reactive cohort and more stable over time. Next, we evaluated CD4^+^ T cell responses against the dominant cross-reactive epitope (iCope) within the fusion domain of spike (aa 816–830). Primary AZ vaccination induced a quantitatively and qualitatively weak response, that was boosted significantly by the second dose with BNT (**Figure 3B**). Compared to the homologous BNT vaccination regimen, heterologous vaccinated individuals exhibited a lower increase of iCope-specific T cells early in the immune response at day 7 and peaked at lower levels following primary immunization (**Figure 3C**). In line, comparison of SI changes between d0 and d7 or d14 revealed an early response of cross-reactive clones upon priming with the BNT vaccine whereas donors vaccinated with AZ responded rather late around d14, indicative of recruitment and expansion of only naïve T cell clones rather than recruitment from a pre-existing cross-reactive repertoire (**Figure 3D**). Compared to primary AZ vaccination, primary BNT vaccination also elicited T cell responses of higher TCR avidity, indicated by higher frequencies of CD3 surface downregulation (CD3^lo^) in activated CD4^+^ T cells. Among the few detectable iCope-reactive T cells in AZ-primed individuals, a larger proportion displayed an *in vivo* activation phenotype (HLA-DR^+^CD38^+^) at d14 compared to BNT-vaccinated donors (**Figure 3C**). In the homologous BNT vaccination regimen, early responses of cross-reactive CD4^+^ T cell clones correlated with higher and more robust antibody titers, as already shown in Loyal et at., 2021 (29). However, we could not identify any correlation between early S-I, S-II or iCope responses (d4, d7) with early and late IgG and IgA titers upon vaccination (first dose d14, second dose d0 or second dose d28) in heterologous vaccinated donors (**Figure S2A**). This lack of measurable early reactivation of cross-reactive CD4^+^ T cells in primary AZ vaccination suggests that this immunization cannot leverage pre-existing T cells to augment the primary response, which was facilitated by BNT primary vaccination and is associated with advantages for both cellular (higher TCR avidity) as well as the humoral (earlier onset) immune response (**Figure S2A**, (29). However, an overall robust IgG and IgA humoral response correlates with a general good S-I but not S-II T cell reactivity later in the response (**Figure S2B**).

### Heterologous AZ-BNT vaccination recruits a distinct antibody repertoire compared to homologous BNT-BNT vaccination

**Figure 4:**
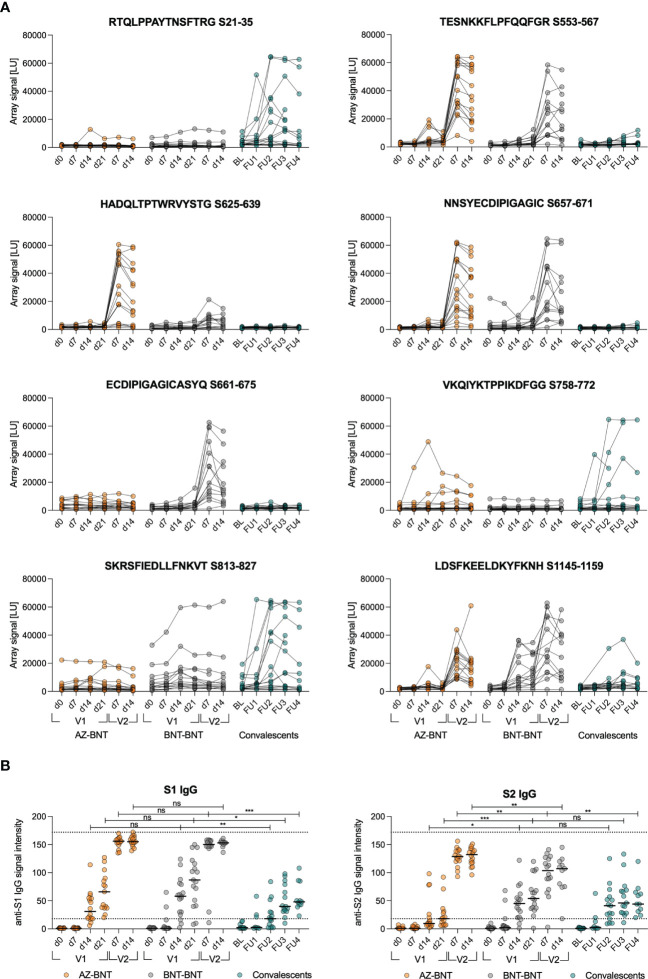
Cross-reactive humoral responses are not effectively induced by AZ primary vaccination. **(A)** Signal from sample incubation on peptide microarrays for selected peptides following AZ-BNT (n=16), BNT-BNT (n=15) vaccination or infection (convalescents (CS), n=17). **(B)** Levels of anti-S1 or anti-S2 IgG binding antibody intensity units in indicated cohorts. Dotted lines indicate lower cut-off at 18 for values classified as positive and upper cut-off at 172. BL=baseline, FU=follow-up. ns=not significant, *P < 0.05, **P < 0.01, ***P < 0.001. Mann-Whitney test. Black line indicates the median.

Next, we compared *de novo* and cross-reactive humoral immune responses in homologous BNT-BNT versus heterologous AZ-BNT vaccination. We screened for linear epitope hot spots of humoral immunity within spike by utilizing a peptide microarray displaying a scan through the spike protein with linear 15mers (overlapping by 11 amino acids (aa)) and calculated the responsiveness per amino acid. In both homo- and heterologous vaccinated cohorts we observed three dominant immunogenic regions: aa 537–609, aa 624–676, and aa 1144–1200 (**Figures 4A**; **S2A, B**). By contrast, convalescents (CS) only responded weakly to a region at aa 19–43 located within the NTD and strongest to two distinct regions between aa 777–829, containing iCope and the fusion domain.

Homologous BNT vaccination induced antibodies only towards the segment of aa809–829, whereas heterologous vaccination resulted in a humoral response linear epitope pattern comparable with natural infection but lower (**Figures 4A**; **S3A, B**). Longitudinal linear peptide-specific antibody analysis revealed poor induction of cross-reactive humoral immunity against the conserved regions aa 813–827 and 1145–1159 by primary AZ vaccination, however, antibodies to the latter were induced by secondary BNT vaccination (**Figures 4A**, **S2B**). Strikingly, AZ-primed donors predominantly reacted against linear epitopes in the S1 part of spike that is less conserved among coronaviruses (**Figures 4A**, **S2A, B**). We also assessed whether the differences in linear epitope recognition was reflected in binding full spike S1 or S2 protein subunits. S1 binding was comparable after infection or primary vaccination with either AZ or BNT. However, S2 binding was comparable only between naturally infected and BNT-primed individuals, while primary AZ vaccination resulted in low levels of anti-S2 antibodies in 8 out of 16 donors (**Figure 4B**).

### T cell clonotype repertoire breadth and depth are comparable in heterologous and homologous vaccination

**Figure 5:**
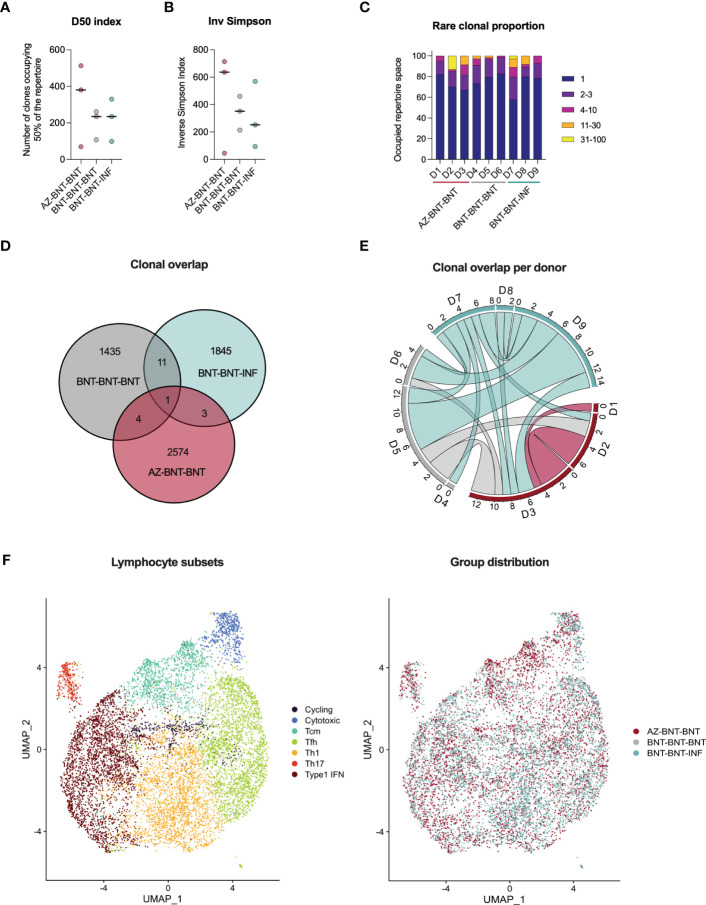
Three months post last antigen contact, single cell RNA sequencing of S-I-reactive CD4+ T cells reveals comparable TCR repertoire in heterologous (AZ-BNT-BNT) vaccinated, homologous (BNT-BNT-BNT) vaccinated and homologous vaccinated and infected (BNT-BNT-INF) individuals. **(A)** D50 index indicates the number of clones occupying 50 % of the repertoire. **(B)** Inverse Simpson Index indicates the TCRαβ repertoire diversity. High values represent a more even distribution of clonotypes, whereas low values indicate enrichment of certain clonotypes. **(C)** Rare clonal proportion shows the summary proportion of clonotypes with specific counts. **(D)** Venn diagram displaying the repertoire overlap between groups. **(E)** Circos plot giving the numbers of shared clones between samples of the different cohort. **(F)** UMAP clustering of seven lymphocyte subsets based on marker genes and distributions of groups across them. **(A, B)** Mann-Whitney test revealed no significant differences between groups (*P* > 0.05). Black line indicates the median.

To assess the capacity of *de novo* recruitment of naive T cells (excluding cross-reactive clones targeting the S-II part) we compared S-I-specific CD40L^+^4-1BB^+^ CD4^+^ T cells of donors undergoing heterologous AZ-BNT-BNT with homologous BNT-BNT-BNT vaccination and additionally to natural infection after BNT-BNT vaccination (BNT-BNT-INF) by droplet scRNA-seq three months post last antigen encounter. Diversity 50 (D50, i.e. the number of dominant clones occupying 50 % of the total repertoire) and inverse Simpson index (38) indicated higher TCR breadth in the heterologous vaccinated, but diversity was overall comparable between groups (**Figures 5A, B**). None of the conditions resulted in a strong enrichment of clonotypes (**Figure 5C**). Despite high diversity between samples, some overlapping TCR clones could be found both between donors of the same group and in-between groups. Here, clonal overlap between BNT-BNT-BNT-vaccinated and BNT-BNT-INF individuals was more than threefold higher than between AZ-BNT-BNT-vaccinated and BNT-BNT-INF individuals (**Figure 5D**). All donors demonstrated overlapping clones, both in absolute and relative (normalized to the sample size) numbers (**Figures 5E**, **S4A**). We also found no difference in the phenotypic distribution of S-I-specific clones across homo- and heterologous vaccinated and vaccination-breakthrough-infection groups indicating a comparable breadth and quality of antigen-specific CD4^+^ T cells in all three groups (**Figures 5F**, **S4B**).

## Discussion

Cross-reactive immunity, resulting from previous exposure to common cold coronaviruses, has been shown to benefit early immune responses during SARS-CoV-2 infection (29–32, 37, 39). We have also shown that primary BNT vaccination engages these pre-existing cross-reactive T cells within the first week following immunization resulting in a more rapid response and higher frequencies of high-quality CD4^+^ T cells compared with donors in whom such pre-existing, cross-reactive T cells were not detectable (29, 40). However, the capacity of vector-based AZ vaccines to elicit cross-reactive immunity was unknown. By comprehensively characterizing a young, healthy cohort of volunteers receiving an AZ-BNT vaccination regimen, we here found slower onset of spike-specific CD4^+^ T cell responses suggesting that pre-existing cross-reactive cellular immunity was not activated when primed with AZ. Moreover, CD4^+^ T cell immunity towards the universal, immunodominant coronavirus-specific epitope iCope (S816-830) was only weakly induced by primary vector-based AZ vaccination compared to primary mRNA-based BNT vaccination, and AZ induced significantly fewer CD4^+^ T cells with high functional TCR avidity. However, following a secondary BNT vaccination, iCope-responsiveness was readily detectable in heterologous immunized individuals. Notably, humoral immunity towards the S1 subunit was comparable between both vaccination regimens, whereas humoral immunity towards the conserved S2 subunit was reduced in AZ-primed individuals. This suggests an altered B cell immune response upon priming with AZ, which was rectified with the secondary BNT vaccination increasing anti-S2 IgG titers to higher levels than homologous BNT-BNT vaccination. Interestingly, spike peptide array analysis revealed distinct areas of humoral epitope recognition, with different profiles depending on the priming vaccine. Our findings are validated by Ng et al., who found AZ vaccination to result in reduced B cell responses targeting distinct S2 regions of spike, especially those against iCope (S816-830) and S1145–1159 (41). It has been shown that iCope-specific antibodies account for 20% of overall neutralization capacity in blood of convalescents, potentially by altering the fusion peptide accessibility (41). S1144–1159 is located in the stem helix and antibodies targeting this area were previously shown to inhibit spike-mediated membrane fusion for beta coronaviruses (42, 43). Both epitopes are immunodominant and highly conserved across human coronaviruses (44, 45) which suggests that the respective areas of spike are probably indispensable for membrane fusion. In addition, antibodies targeting the S2-located, highly conserved HR1 domain were shown to act as pan-coronavirus fusion inhibitor (46, 47). Accordingly, antibodies directed against these regions could play a critical role in protecting against SARS-CoV-2 and other coronavirus infections and hence in disease control. The search for vaccination regimens that could specifically boost these antibodies might be important for future pandemics involving novel coronavirus VOCs.

The significantly higher antibody titers observed following heterologous vaccination may be attributed to the three-month time window between first and second vaccine dose (AZ-BNT), allowing for prolonged germinal center maturation, resulting in high affinity maturation of previously low to non-binding antibodies (48, 49). AZ-AZ vaccination with a three month interval was reported to mount lower titers than observed here and BNT-BNT vaccination with a three week interval induced comparable titers in relation to heterologous vaccination (13, 14, 50). This suggests that other mechanisms may be responsible for the lower titers in homologous AZ prime-boost vaccination regimens, such as vector immunity (48, 49). Additionally, secondary BNT-immunization containing a slightly different spike protein variant than the AZ vaccine exposed the B cells to new epitopes not yet covered by AZ-induced antibodies. However, while mutations in neutralizing epitopes comparatively affect neutralization capacity in homo- and heterologous vaccinated donors, homologous BNT vaccination shows higher neutralization capacity against immune escape variant Omicron BA.5 after the third dose. This finding highlights the importance of in-depth understanding of the underlying immune maturation mechanisms to design long-term protective vaccines and vaccination schedules.

The observed differences in linear spike epitope antibody coverage between AZ and BNT vaccination may originate from structural differences of the spike protein expressed during the distinct vaccination regimens. Underlying mechanisms could be sequence modifications, different processing in target cells (receptor mediated uptake in vector vaccines, random uptake in lipid nanoparticle encapsulated mRNA vaccines), different glycosylation patterns and protein stability. The transmembrane protein consists of three S2 subunits with three non-covalently attached S1 subunits (51). The N-terminal S1 subunit harbors the N-terminal domain (NTD) and receptor binding domain (RBD) and is relevant for ACE2 recognition on the host cell. Upon binding of ACE2, proteolytic cleavage at the S2’ site results in the dissociation of S1 and a conformation change of S2 into post-fusion conformation which facilitates the membrane fusion (51–54). The fusion peptide region harboring iCope is concealed by S1 in the pre-fusion status but becomes exposed upon S2’ cleavage and S1 dissociation until it penetrates the host membrane in the post-fusion conformation (55). Spontaneous S1 shedding has been described to occur in non-stabilized spike, whereas two Prolines introduced in S2 at K986P and V987P in BNT162b2 as well as Moderna’s mRNA-1273 vaccine prevent a conformational switch into an elongated alpha helix of the post-fusion form (56, 57). For both mRNA vaccines, the recruitment of pre-existing, cross-reactive immunity into the immune response was previously shown (29, 37). Therefore, lack of stabilizing mutations in AZ might result in an altered accessibility to the fusion-peptide-derived epitopes. Whether and how this changed conformation then translates into an apparently less efficient priming of cross-reactive B and T cells remains to be elucidated. We also observed delayed induction of cellular immune responses in AZ vaccinees relative to primary BNT vaccination. This may be due to the fact that the lipid nanoparticle-formulated, nucleoside-modified delivered mRNA could be rapidly available to the immune system, whereas vector-based antigen delivery additionally requires infection of the host cell and transcription of the adenoviral DNA (58). At day 14, the differences became smaller.

Our findings are limited by the lack of further cohorts, particularly a homologous AZ vaccination and a homologous BNT vaccination group with a three-month interval as control group, as well as relatively small cohorts for the kinetics and the single cell RNA-sequencing. Additionally, it would be interesting to check for differences in the TCR repertoire following only primary vaccination and against the whole spike protein. Finally, comparison of other pre-fusion stabilized versus non-stabilized vaccines would prove the concept of optimal pan-coronavirus immunity induction by stabilized variants independently of their mRNA versus vector basis. To address the constant challenges of newly arising VOCs, second generation vaccines should target pan-coronavirus immunity, focusing on conserved regions and ideally activating mutation-resilient immunity. We here demonstrate that the AZ vector vaccine induces robust adaptive immune responses however does not engage cross-reactive pan-coronavirus immunity targeting the conserved S2 subunit of spike.

## Materials and methods

### Study participants

This study was approved by the Institutional Review board of the Charité (EA/152/20). Written informed consent was obtained from all included participants and the study was conducted in agreement with the declaration of Helsinki. All vaccinated donors were assessed for age and gender as indicated in **Supplementary Table 1**. The timepoints week 18 (w18) after second and week 12 (w12) after the third dose of vaccine spans the days 131-165 and 85-126 respectively. Previous infection was excluded by a questionnaire asking for SARS-CoV-2 related symptoms and nucleocapsid IgG ELISA. Detailed specifications of the convalescent cohort including the time points of the follow-up measurements (FU) and symptoms are given in Loyal et al., Science, 2021 (29).

### Coronavirus RT-qPCR

For all visits and donors, RNA was extracted from 140 μl of wet nasopharyngeal swabs (Copan mini UTM) using the QIAamp Viral RNA Mini Kit and QIAcube Connect with the manual lysis protocol. SARS-CoV-2 RNA detection was performed using a simultaneous two duplex one-step real-time RT-PCR assay with primers and probes (in-house protocol, primers and probes ordered at Metabion and Thermo Fischer Scientific (MGB probe)) for SARS-CoV-2 E Gene and SARS-CoV-2 ORF1ab according to the RKI/ZBS1 SARS-CoV-2 protocol as described before (59). Each one is duplexed with a control that either indicates potential PCR inhibition or proves the successful extraction of nucleic acid from the clinical specimen. As positive controls genomic SARS-CoV-2 RNA and genomic SARS-CoV RNA were used for the ORF1ab and the E-Gene assay, respectively, adjusted to the Ct values 28 and 32. PCR was conducted with the AgPath-ID™ One-Step RT-PCR Reagents kit (Applied Biosystems) using a Bio-Rad CFX96 or Bio-Rad Opus real-time PCR cycler.

### SARS-CoV-2 IgG and IgA S1 ELISA

Anti-SARS-CoV-2 IgG and IgA ELISA specific for the S subunit 1 (S1) was performed using the commercial kits (QuantiVac for IgG), EUROIMMUN Medizinische Labordiagnostika AG) according to the manufacturer’s instructions. Upper and lower cut-off were set at a ratio of 1 and 13 for IgG, respectively, and at 1 and 10 for IgA, respectively.

### Epitope-specific antibody ELISA

400nM of biotinylated peptide S809-826 (Biotin-Ttds-PSKPSKR*SFIEDLLFNKV*-OH, Ttds linker: N-(3-{2-[2-(3-Amino-propoxy)-ethoxy]-ethoxy}-propyl)-succinamic acid, JPT Peptide Technologies) was immobilized on a 96-well Streptavidin plate (Steffens Biotechnische Analysen GmbH) for 1 hour at RT. After blocking (1 hour, 30°C) serum samples were diluted 1:100 and incubated for 1 hour at 30°C. HRP-coupled, anti-human-IgG secondary antibody (Jackson Immunoresearch) was diluted 1:5000 (Jackson Immunoresearch) and added to the serum samples for 1 hour at 30°C, then HRP substrate was added (TMB, Kem-En-Tec). The reaction was stopped by adding sulfuric acid and absorption was measured at 450 nm using a FlexStation 3.

### SARS-CoV-2 spike epitope-specific peptide microarray

The peptides were synthesized using SPOT synthesis, cleaved from the solid support and chemoselectively immobilized on functionalized glass slides. Each peptide was deposited on the microarray in triplicates. The peptide microarrays were incubated with human sera (applied dilution 1:200) in a 96-well microarray incubation chamber for one hour at 30°C, followed by incubation with 0.1 μg/ml fluorescently labeled anti human IgG detection antibody (Jackson Immunoresearch). Washing steps were performed after each incubation step with 0.1 % Tween-20 in 1x TBS. After the final incubation step the microarrays were washed and dried. Each microarray slide was scanned using a GenePix Scanner 4300 SL50 (Molecular Devices). Signal intensities were evaluated using GenePix Pro 7.0 analysis software (Molecular Devices). For each peptide, the MMC2 value of the three triplicates was calculated. The MMC2 value was equal to the mean value of all three instances on the microarray except when the coefficient of variation (CV) – standard-deviation divided by the mean value – was larger than 0.5. In this case the mean of the two values closest to each other (MC2) was assigned to MMC2. Further data analysis and generation of the bar plots was performed using the statistical computing and graphics software R (Version 4.1.1, (60)).

### SARS-CoV-2 pseudovirus neutralization assay

The SARS-CoV-2 pseudovirus neutralization assay was conducted as previously described in (61). Shortly, SARS-CoV-2 pseudoviruses were generated by co-transfection of plasmids encoding HIV Tat, HIV Gag/Pol, HIV Rev, luciferase followed by an IRES and ZsGreen, and the alpha and omicron BA.5 SARS-CoV-2 spike protein into HEK 293T cells using FuGENE 6 Transfection Reagent (Promega). Virus culture supernatant was harvested at 48 h and 72 h post transfection and stored at -80°C till use. Harvested virus was titrated by infecting 293T expressing ACE242 and after a 48-hour incubation at 37°C and 5 % CO_2_, luciferase activity was determined after addition of luciferin/lysis buffer (10 mM MgCl2, 0.3 mM ATP, 0.5mM Coenzyme A, 17 mM IGEPAL (all Sigma-Aldrich), and 1 mM D-Luciferin (GoldBio) in Tris-HCL) using the Tristar microplate reader (Berthold). Neutralization assays were performed as described before. Briefly, 3-fold serial dilutions of serum (1:10 starting dilution) were co-incubated with pseudovirus supernatants for 1 h at 37°C, following which 293T-ACE-2 cells were added. After 48 h at 37°C and 5% CO_2_, luciferase activity was determined using the luciferin/lysis buffer. Background relative light units (RLUs) of non-infected cells was subtracted and 50% inhibitory dilution (ID_50_) were calculated as the serum dilution resulting in a 50% reduction in RLU compared to the untreated virus control wells. ID_50_ values were calculated by plotting a non-linear fit dose response curve in GraphPad Prism 7.0.

### Blood and serum sampling and PBMC isolation

Whole blood was collected in lithium heparin tubes for peripheral blood mononuclear cells (PBMC) isolation and SST™II advance (Vacuette^®^, Greiner Bio One and Vacutainer BD) tubes for serology. SST™II advance tubes were centrifuged for 10 min at 1000 g prior to removing serum. Serum aliquots were frozen at -20°C until further use. PBMCs were isolated by gradient density centrifugation according to the manufacturer’s instructions (Leucosep tubes, Greiner; Biocoll, Bio&SELL).

### *Ex vivo* T cell stimulation

Freshly isolated PBMC were cultivated at a concentration of 5*10^6^ PBMC/ml in AB-medium containing RPMI 1640 medium (Gibco) supplemented with 10 % heat inactivated AB serum (Pan Biotech), 100 U/ml of penicillin (Biochrom), and 0.1 mg/ml of streptomycin (Biochrom). Stimulations were conducted with PepMix™ overlapping peptide pools (15 aa length with 11 aa overlaps, JPT Peptide Technologies) covering the proteins of interest (all JPT Peptide Technologies). iCope single peptide stimulation was conducted with iCope (N’-SFIEDLLFNKVTLAD-C’ (all JPT Peptide Technologies)). All stimulations (peptide pools and single peptides) were performed at final concentrations of 1 µg/ml per peptide. For negative control the stimulation peptide solvent DMSO diluted 1:1 in PBS was used at the same concentration as in peptide-stimulated tubes. The CEFX Ultra SuperStim pool (1 µg/ml per peptide) (JPT Peptide Technologies) was used as positive stimulation control. For optimized costimulation, purified anti-CD28 (clone CD28.2, BD Biosciences) was added to each stimulation at a final concentration of 1 µg/ml. Incubation was performed at 37°C, 5% CO_2_ for 16 hours in the presence of 10 µg/ml Brefeldin A (Sigma-Aldrich) during the last 14 hours. CD4^+^ T cell activation was calculated as a stimulation index (SI). Stimulation index is the ratio of CD40L^+^4-1BB^+^ CD4^+^ T cells in the stimulation to the percentage of CD40L^+^4-1BB^+^ CD4^+^ T cells in the unstimulated control. Stimulation index between 1.5 and 3 indicate a response with uncertainty, an index of 3 and higher a definite response. Both limits are indicated by dotted lines in the respective figures.

### Flow cytometry

Stimulations were stopped by incubation in 2mM EDTA for 5 min. Surface staining was performed for 15 min in the presence of 1 mg/ml of Beriglobin (CSL Behring) with the following fluorochrome-conjugated antibodies titrated to their optimal concentrations as specified in **Supplementary Table 2**: anti-CD3-FITC (Miltenyi), anti-CD4-VioGreen (Miltenyi), anti-CD8-VioBlue (Miltenyi), anti-CD38-APC (Miltenyi), and anti-HLA-DR-PerCpVio700 (Miltenyi). During the last 10 min of incubation, Zombie Yellow fixable viability staining (Biolegend) was added. Fixation and permeabilization were performed with eBioscience™ FoxP3 fixation and PermBuffer (Invitrogen) according to the manufacturer’s protocol. Intracellular staining was carried out for 30 min in the dark at room temperature with anti-4-1BB-PE (Miltenyi), anti-CD40L-PEVio770 (Miltenyi) and anti-CD40L-PECy7 (Biolegend), anti-IFN-γ-A700 (Biolegend) and anti-TNF-α-BV605 (Biolegend). All samples were measured on a MACSQuant^®^Analyzer 16 (Miltenyi). Instrument performance was monitored prior to every measurement with Rainbow Calibration Particles (BD Biosciences).

### Single-cell RNA sequencing

For single-cell RNA sequencing, PBMC of three BNT-BNT-BNT-vaccinated, three AZ-BNT-BNT vaccinated, and three BNT-BNT-infected donors were stimulated with 1 µg/ml S-I peptide pool in the presence of purified anti-CD28 (clone CD28.2, BD Biosciences) and anti-CD40 (clone HB14, Miltenyi Biotec). CD4^+^ T cells were enriched by MACS (Miltenyi Biotec) and CD40L^+^4-1BB^+^ CD4^+^ T cells FACS sorted using an FACS Melody (BD). The cells were loaded with a maximum concentration of 1000 cells/µl and a maximum cell number of 17.000 cells on a Chromium Chip G (10x Genomics). Gene expression and TCR libraries were generated according to the manufacturer’s instruction using the Chromium Next GEM single cell 5’Library and Gel bead Kit V1.1 and Chromium Single Cell V(D)J Enrichment Kit for human T cells (10x Genomics). Sequencing was conducted with a NovaSeq 6000 cartridge (Illumina) with 20.000 reads per cell for GEX libraries and 5.000 reads per cell for TCR libraries.

### Single-cell transcriptome analysis

Single cell RNA expression data were mapped to reference genome GRCh38-2020-A and preprocessed using the Cell Ranger Software v6.1.2 (10x Genomics). Quality control and analysis of data was done in R 4.0.5 (R Core Team (2021). R: A language and environment for statistical computing. R Foundation for Statistical Computing, Vienna, Austria. URL https://www.R-project.org/) using the “Seurat” package (62). To remove low quality cells, doublets and empty cells thresholds were set to 840–4000 RNA features and less than 5 % mitochondrial RNA per cell. Data were normalized by using the LogNormalize function of the Seurat package and genes detected in less than 0.1% of the cells were excluded. For gene expression analysis the TCR genes were excluded from the data set to avoid TCR biased clustering. A heatmap with the scaled expression values of selected genes was generated using the DoHeatmap() function of the Seurat package. Furthermore, the expression values of these genes were aggregated according to the experimental groups and shown in a heatmap generated with GraphPad Prism.

### Single cell TCR analysis

Single cell TCR data were preprocessed using the Cell Ranger Software v6.1.2 (10x Genomics) and the GRCh38-2020-A reference genome. Data was further processed in R using the “immunarch” package (63). Only cells which passed the quality controls in the gene expression analysis and containing exactly one TCR alpha and one TCR beta chain were used for further analysis. D50 Index, Inverse Simpson Diversity index and rare clonal proportions were calculated using the corresponding functions of the immunarch package. Overlaps of clonotypes between experimental groups were determined using the repOverlap function and visualized as heatmap and with the vis_circos functions of the immunarch package. Numbers for the Venn diagram were calculated using the calculate.overlap function of the VennDiagram package Version 1.7.3 (64).

### Data analysis and statistics

Study data were collected and managed using REDCap electronic data capture tools hosted at Charité (65, 66). Flow cytometry data were analyzed with FlowJo 10.6 (FlowJo LLC), and statistical analysis conducted with GraphPad Prism 9. If not stated otherwise, data are plotted as median. *N* indicates the number of donors. *P*-values were set as follows: *P <0.05, **P <0.01, and ***P<0.001.

## Data availability statement

Raw single-cell sequencing data is available in the GEO repository, accession number GSE222872.

## Ethics statement

The studies involving human participants were reviewed and approved by Institutional Review board of the Charité (EA/152/20). The patients/participants provided their written informed consent to participate in this study.

## Author contributions

Conceptualization: LH, LL, and AT; Data curation: LH and JB; Formal Analysis: LH; Funding acquisition: AT; Investigation: LH, JB, and LL; Resources: LM-A, KJ, MS, JM, MG, MM, MD, BK, PH, NM, UR, ME, KS, HW, BT, FK and AN; Visualization: LH, JB, KJ, PH and LL; Writing: LH, CG-T, LL and AT. All authors contributed to the article and approved the submitted version.
