# Supplementary material for: Primary ChAdOx1 vaccination does not reactivate pre-existing, cross-reactive immunity
Source: Front Immunol. 2023 Jan 31;14:1056525. doi: 10.3389/fimmu.2023.1056525 (PMC9927399; doi:10.3389/fimmu.2023.1056525)
Supplement: Supplementary file 3 [file DataSheet_1.pdf]

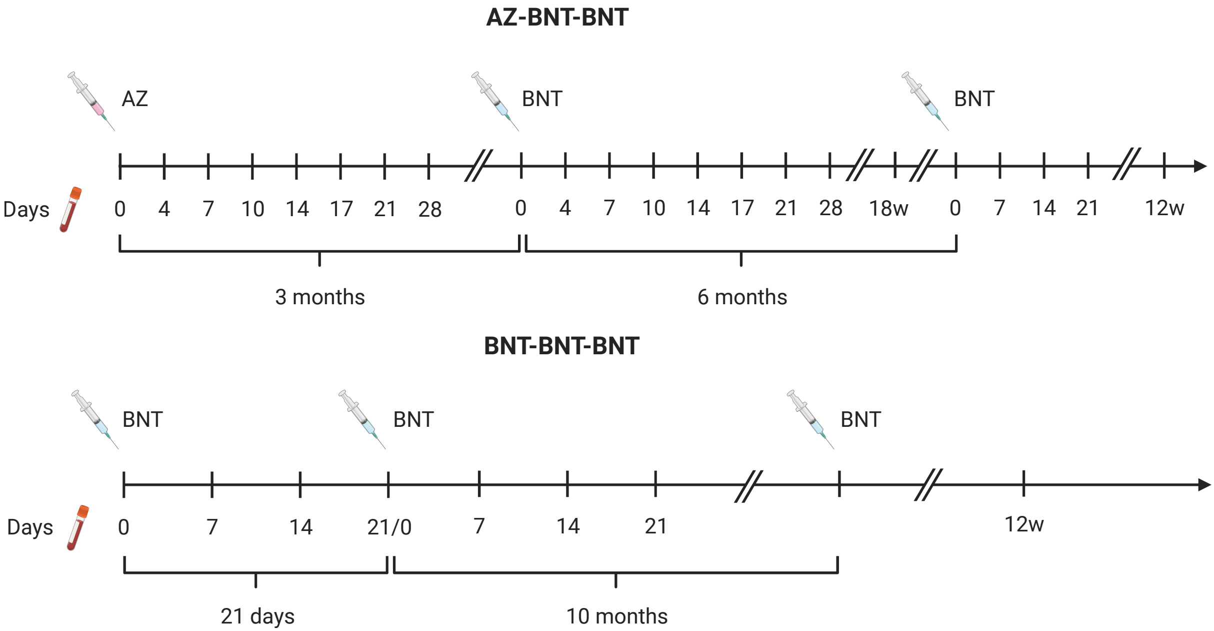

Figure S1: Timeline of heterologous and homologous vaccination and study sampling events. Syringes mark days of vaccine administration (AZ: ChAdOx1; BNT: BNT162b2). Blood was drawn prior to first vaccination (day0) and on indicated days or weeks (18w, 12w) post last vaccination event. Created with BioRender.com.
