# Supplementary material for: Primary ChAdOx1 vaccination does not reactivate pre-existing, cross-reactive immunity
Source: Front Immunol. 2023 Jan 31;14:1056525. doi: 10.3389/fimmu.2023.1056525 (PMC9927399; doi:10.3389/fimmu.2023.1056525)
Supplement: Supplementary file 4 [file DataSheet_2.pdf]

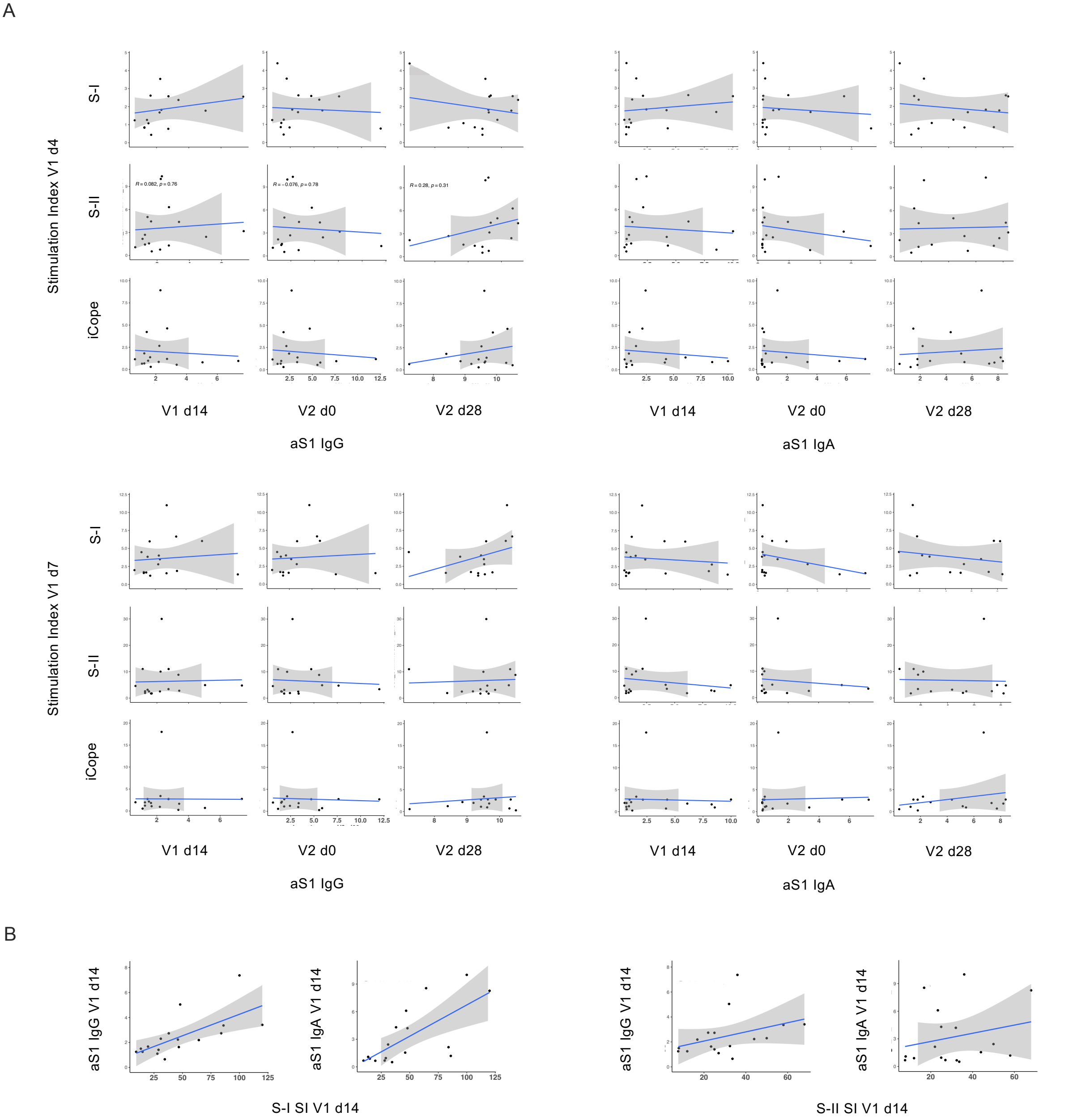

Figure S2: Correlations of humoral and cellular immune response. (A) Correlation of early (d4, d7) S-I, S-II and iCope stimulation index with IgG and IgA antibody titers at peak of response (d14), prior to secondary vaccination (V2 d0), and 28 days post secondary vaccination (V2 d28) determined by ELISA. **(B)** Correlation of S-I and S-II stimulation index with IgG and IgA level determined by ELISA at peak of immune response after primary AZ vaccination.
