# Supplementary figures and images for: Primary ChAdOx1 vaccination does not reactivate pre-existing, cross-reactive immunity
Source: Front Immunol. 2023 Jan 31;14:1056525. doi: 10.3389/fimmu.2023.1056525 (PMC9927399; doi:10.3389/fimmu.2023.1056525)

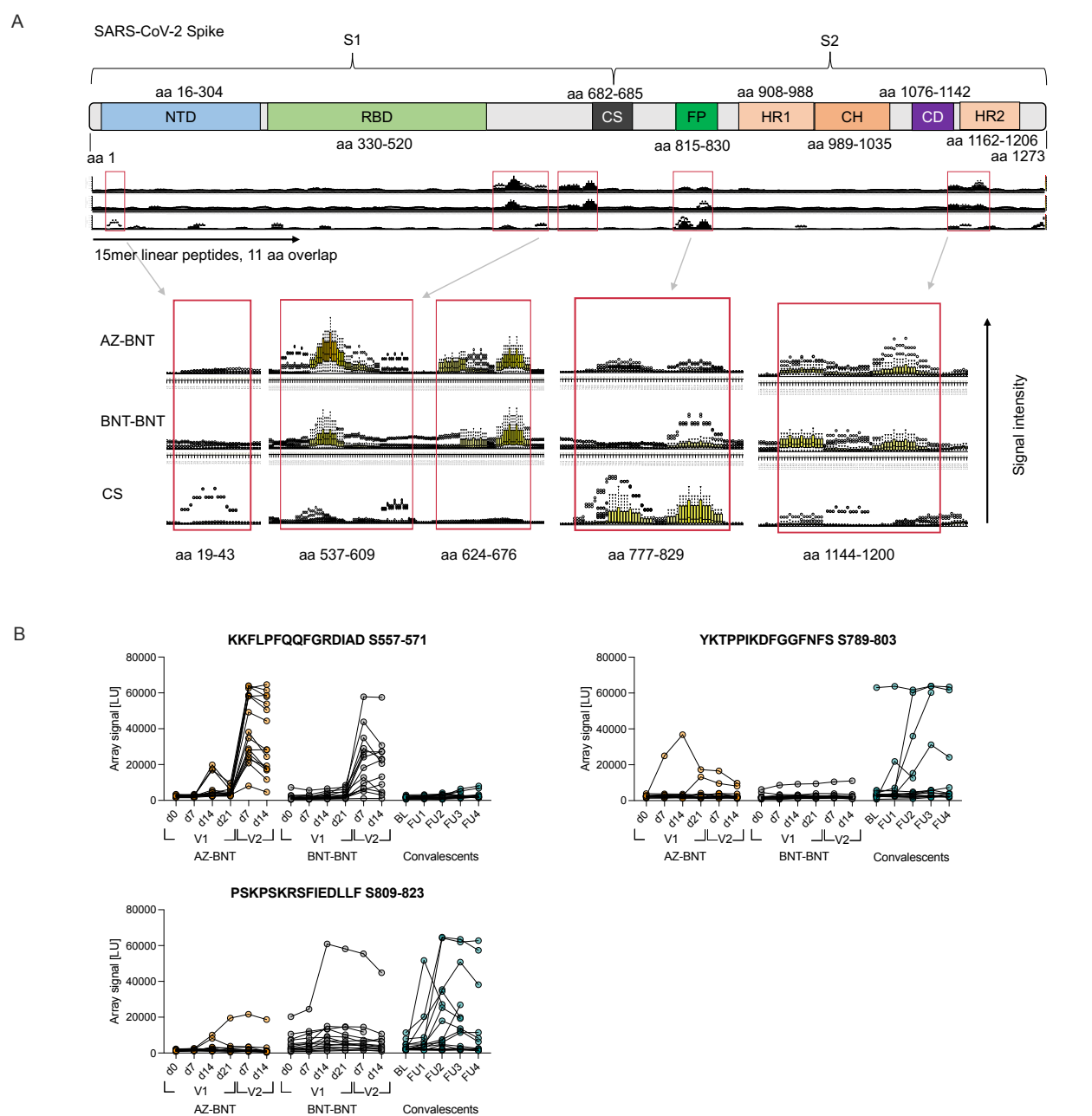

Supplement: Supplementary file 5 [file DataSheet_3.pdf]
