# Supplementary material for: Primary ChAdOx1 vaccination does not reactivate pre-existing, cross-reactive immunity
Source: Front Immunol. 2023 Jan 31;14:1056525. doi: 10.3389/fimmu.2023.1056525 (PMC9927399; doi:10.3389/fimmu.2023.1056525)
Supplement: Supplementary file 6 [file DataSheet_4.pdf]

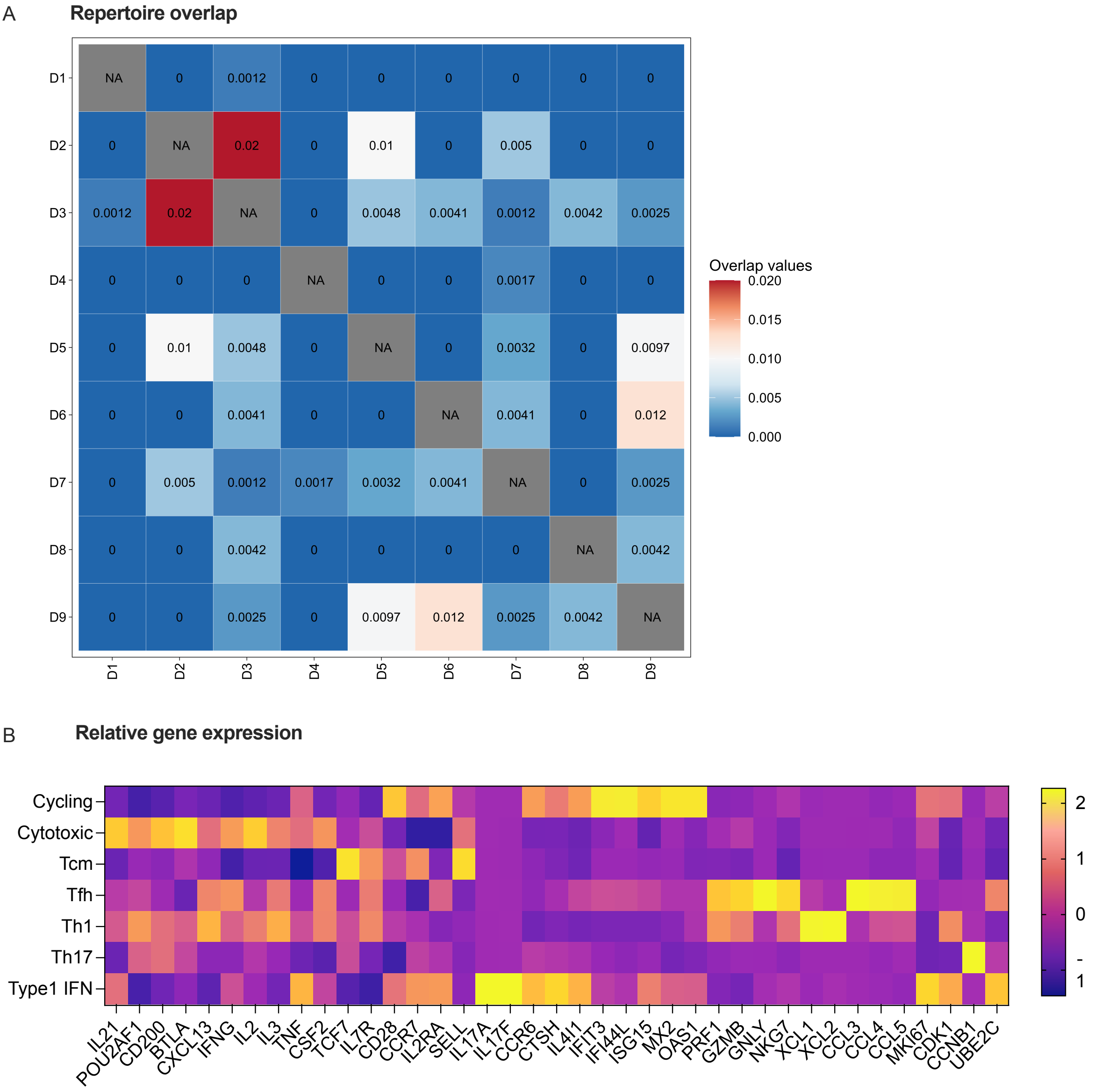

Supplementary Figure 4: Homologous vaccination, heterologous vaccination and vaccination infection result in comparable S-I-specific CD4$^+$ TCR repertoire and phenotype. (A) A normalized measure of TCRab overlap, defined as the size of the intersection divided by the smaller size of the two sets. D1-D3: AZ-BNT-BNT; D4-D6: BNT-BNT-BNT; D7-D9: BNT-BNT-INF **(B)** Relative gene expression of selected marker genes in S-I-specific CD4$^+$ T cells underlying the allocated clusters of Fig.5 F.
